# Bibliographical Notices

**Published:** 1838-01-03

**Authors:** 


### BIBLIOGRAPHICAL NOTICES.

SURGICAL OBSERVATIONS ON TUMORS, *With cases and operations, by* JOHN C. WARREN, *M. D., Professor of Anatomy and Surgery in Harvard University, and Surgeon to the Massachusetts General Hospital.* Sixteen colored plates, 8vo. pp. 607. Boston: CROCKER and BREWER, 1837.

This is not Dr. Warren's first attempt towards enriching the literature of his profession. In 1809 he published a work upon the organic diseases of the heart; at that time the only treatise in the English language, on that important, and then almost unrecognized class of afflictions. This embodied the new views taught by Corvisart, whose book on the same subject, had appeared in Paris three years previously; but which, from the immense difficulties of communication, at that period, between Great Britain and the Continent, was but imperfectly known. This effort, at the onset of a career, since eminently and justly prosperous, was attended with entire success, and secured for the author a considerable reputation, both at home and abroad.

On the present occasion Dr. Warren has produced a work of sterling value, abounding in details inestimable to a practical man. His situation, as Surgeon to the Massachusetts General Hospital, afforded opportunities for the observation and investigation of disease, rarely enjoyed. The volume before us is the result of an unlimited experience, with a description of affections as formidable as frequent. Few subjects are of more vital importance, or demand more cautious consideration, than tumors. Their repeated occurrence, alarming nature, insidious progress, and frequently fatal termination, require the fullest and most accurate knowledge of their character. When we reflect that, oftentimes, upon the promptness and correctness of the diagnosis, the life of the patient may depend, the imperative necessity of an ample and varied acquaintance with their history, must be acknowledged by all "I have known," says Professor Warren, "a tumor to remain free from the least mark of malignity for thirty years, and then to become so, without any imaginable or conceivable cause, in a perfectly healthy subject in the vigor of life." p. 13. To tamper with, or neglect, as trifling, the most insignificant tumor, endangers then the life of the sufferer, and justly exposes the culprit to the severest reprobation, for his ignorance and carelessness; and yet from these very causes, how frequently are valuable lives risked, and even sacrificed.

As a complete and finished monograph upon tumors, this volume can claim no pretensions. It is simply a record of cases, embodying the innumerable varieties of these abnormal productions, with a series of brilliant operations, by which most of them were removed. "It will be understood that this work is not a treatise comprehending all that is known on the subject, but rather as a collection of cases intended to illustrate the distinctions between different tumors." Preface, p. vi.

It is much to be regretted that Dr. Warren was not enabled to bestow more time upon the preparation of this work. As it is, we have little of the natural history of tumors, few pathological remarks, and no analysis of the facts. This is a serious void, which we hope to see filled at some subsequent period, when more leisure, and better health is enjoyed by the ingenious author. The mass of information contained in the multifarious cases presented to the reader, could, we think, be advantageously re-cast, and offered in a more valuable and attractive form. The style is in general, accurate and perspicuous, although occasionally unnecessarily diffuse and familiar. This arises from the preservation of the colloquial diction of the lecturer, and a retention of the slovenly phraseology of the case-book. It is illustrated by sixteen neatly colored engravings. Its *getting up* reflects infinite credit on the enterprising publishers.

We regret that our limits will not permit us to enter into any detailed analysis of the book before us; all that at this time we shall be enabled to do, will be to present a brief outline of the author's plan; at the same moment assuring the profession it will richly reward an attentive perusal.

As the basis of a classification, Dr. Warren has presented "the different tumors under the head of the different textures of the body, as far as may be done." Important and valid objections can be raised against this arrangement; although, upon the whole, it is as satisfactory and practical as any other division of the subject.

"Instead of beginning with the general textures, we shall consider it convenient to speak first of those which are most superficial, most easy of inspection, and whose affections are most simple. Beginning then with the textures of the skin, we take those next, of the cellular membrane, then the muscular, the fibrous and the osseous. After these most obvious and extensive textures, we shall place the numerous tumors of the glandular system; next, those of the vascular; and then those of the membranous textures, mucous, serous, fibrous, and synovial. Finally, tumors composed of different textures, and not found exclusively in any—the encysted tumors. Some remarks will further be made, with a view to direct your attention to the diagnosis of tumors in the cavity of the abdomen." p. 14.

Upon this basis, Professor Warren developes his views, by the introduction of a number of graphic and highly interesting cases elucidative of this important division of surgery.

No one can read this essay without an increased estimate of the skill, knowledge, and abilities of the distinguished author; and an earnest wish that he may be permitted long to enjoy the results of a well-earned and well-deserved reputation.

---

THE AMERICAN MEDICAL LIBRARY AND INTELLIGENCER; *a concentrated record of Medical Science and Literature, edited by* ROBLEY DUNGLISON, *M. D., Professor of the Institutes of Medicine in Jefferson Medical College.* Published semi-monthly: ADAM WALDIE, Philadelphia.

This description of publication exercises, we think, the most salutary influence upon the profession at large. It is a mortifying fact, that but too frequently, the libraries of distant practitioners are composed solely of the text books, which, on the completion of their initiatory studies, they carry home with them. Narrow views, and limited knowledge, are the necessary consequences. The progress of medical science is arrested, and the community are eventually the sufferers. The difficulties of transportation, and the enormous price of medical works, are, in general, the main causes of this deplorable state of things. Dr. Dunglison's plan meets these difficulties. His re-prints place in the hands of the physician, dwelling upon the frontier, the recent medical publications, at an expense trifling compared with their intrinsic value, and at almost as early a period as they are received by his brethren of the Atlantic cities. This must be followed by the happiest effects upon the state of medicine throughout the country.

The American Medical Library has been in operation about eight months, and its subscribers are already in possession of twenty re-prints, all selected with judgment. Among these we may notice Stokes' Theory and Practice of Medicine, a work of sterling value, and without comparison, the best of its description.

